# Supplementary material for: Recurrent structural variation and recent turnover at the 17q21.31 locus in humans and great apes
Source: Nat Commun. 2026 May 19;17:6568. doi: 10.1038/s41467-026-73174-1 (PMC13381761; doi:10.1038/s41467-026-73174-1)
Supplement: Supplementary file 1 — Supplementary Information [file 41467_2026_73174_MOESM1_ESM.pdf]

Figure S1

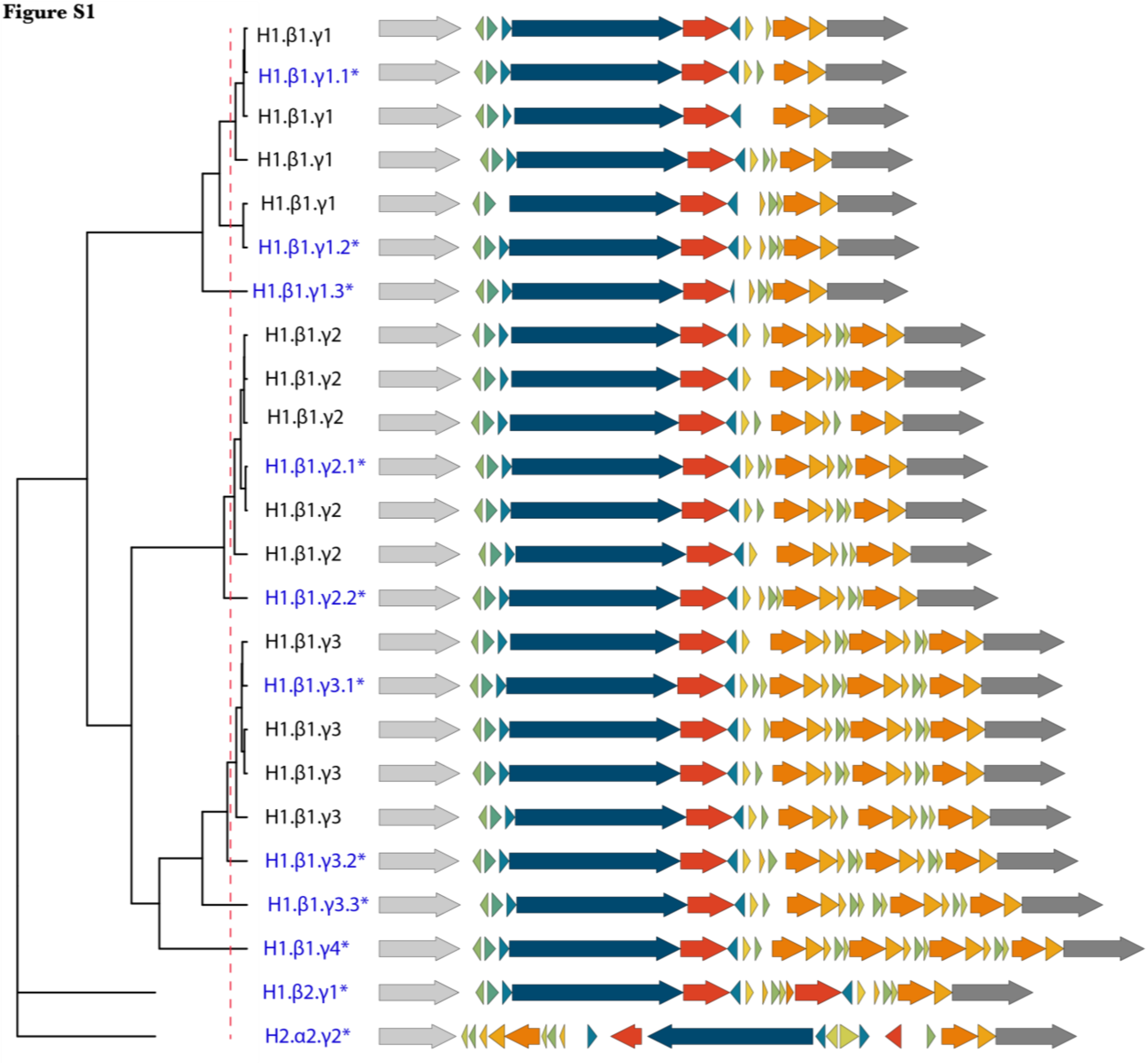

Supplementary Figure 1 – 24 distinct 17q21.31 structural haplotypes from human long read genome assemblies: Schematic of 24 unique structural haplotypes found within 210 long-read haplotype-resolved assemblies. Each row represents a unique structural haplotype. Structural haplotypes are arranged using hierarchical clustering based on jaccard distance, with tip sizes scaled by the number of assemblies sharing each haplotype. The dashed red line indicates where clades were collapsed into a single representative structure as shown in **Figure 1B**.

Figure S2

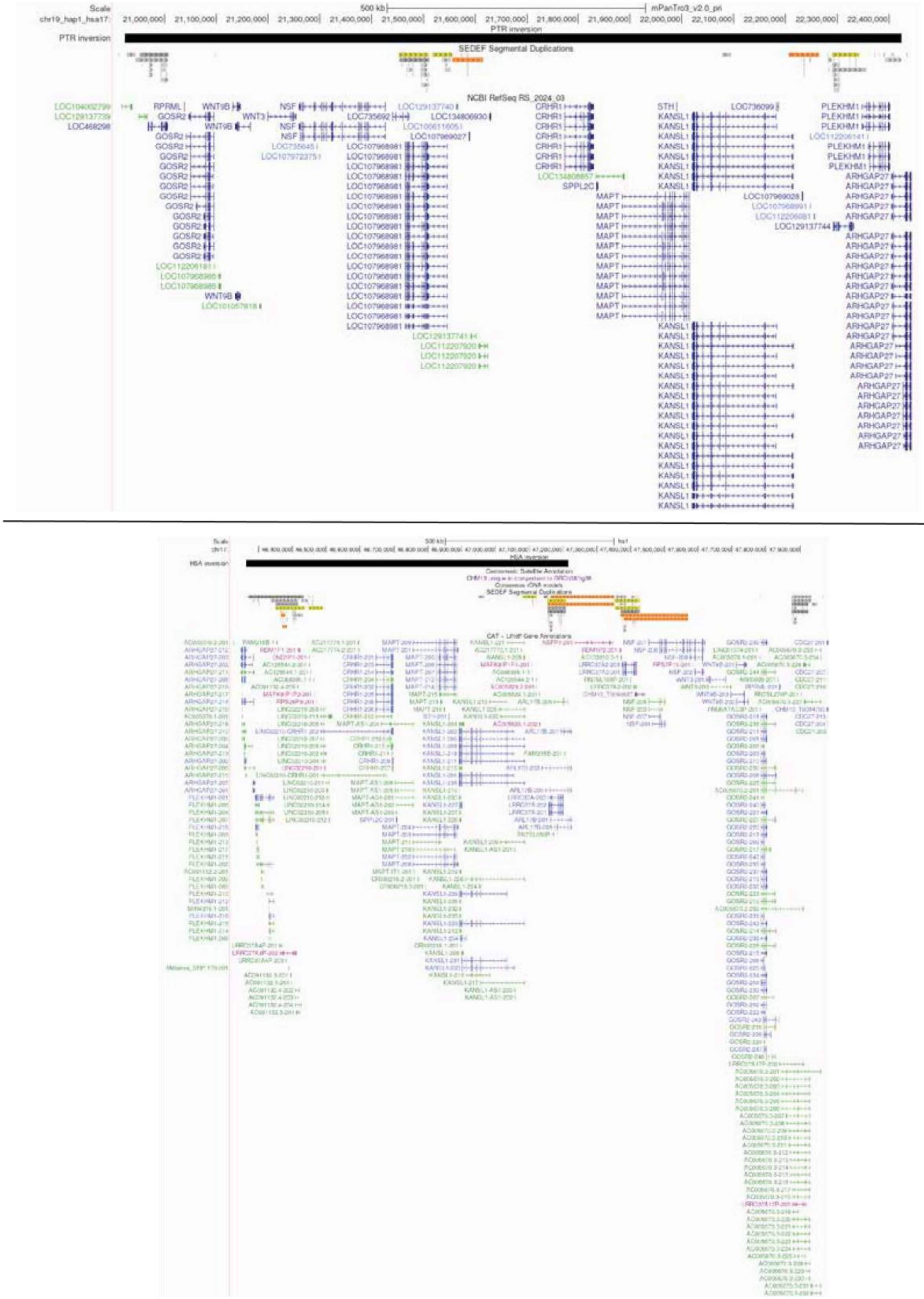

Scale
500 kb
mPanTro3_v2.0_pri
chr19_hap1_hsa17:
21,000,000 | 21,100,000 | 21,200,000 | 21,300,000 | 21,400,000 | 21,500,000 | 21,600,000 | 21,700,000 | 21,800,000 | 21,900,000 | 22,000,000 | 22,100,000 | 22,200,000 | 22,300,000 | 22,400,000
PTR inversion
SEDEF Segmental Duplications
NCBI RefSeq RS_2024_03
RPRML
WNT9B
NSF
GOSR2
WNT3
LOC735692
LOC134806930
CRHR1
STH
LOC736099
PLEKHM1
KANSL1
LOC107968981
SPPL2C
MAPT
ARHGAP27
LOC107969028
LOC129137744
LOC129137741
LOC112207920
Scale
500 kb
hs1
chr17
HSA inversion
SEDEF Segmental Duplications
CAT + Liftoff Gene Annotations

Supplementary Figure 2 - Comparative gene organization and segmental duplications at the 17q21.31 locus in humans and chimpanzees: (Top) Gene names and orientation across the human reference (hg38) 17q21.31 inversion locus and extended distal flanking sequence. The black rectangle denotes the inversion breakpoints of the 17q21.31 locus; orange and yellow rectangles denote the segmental duplications; protein coding genes are shown in blue; non-coding transcripts are shown in green; and pseudogenes are shown in purple. (Bottom) Orthologous inversion in chimpanzees shows a larger inversion region (by size) and additional genes in the chimpanzee region that are not shown in the inversion region in humans.

Figure S3

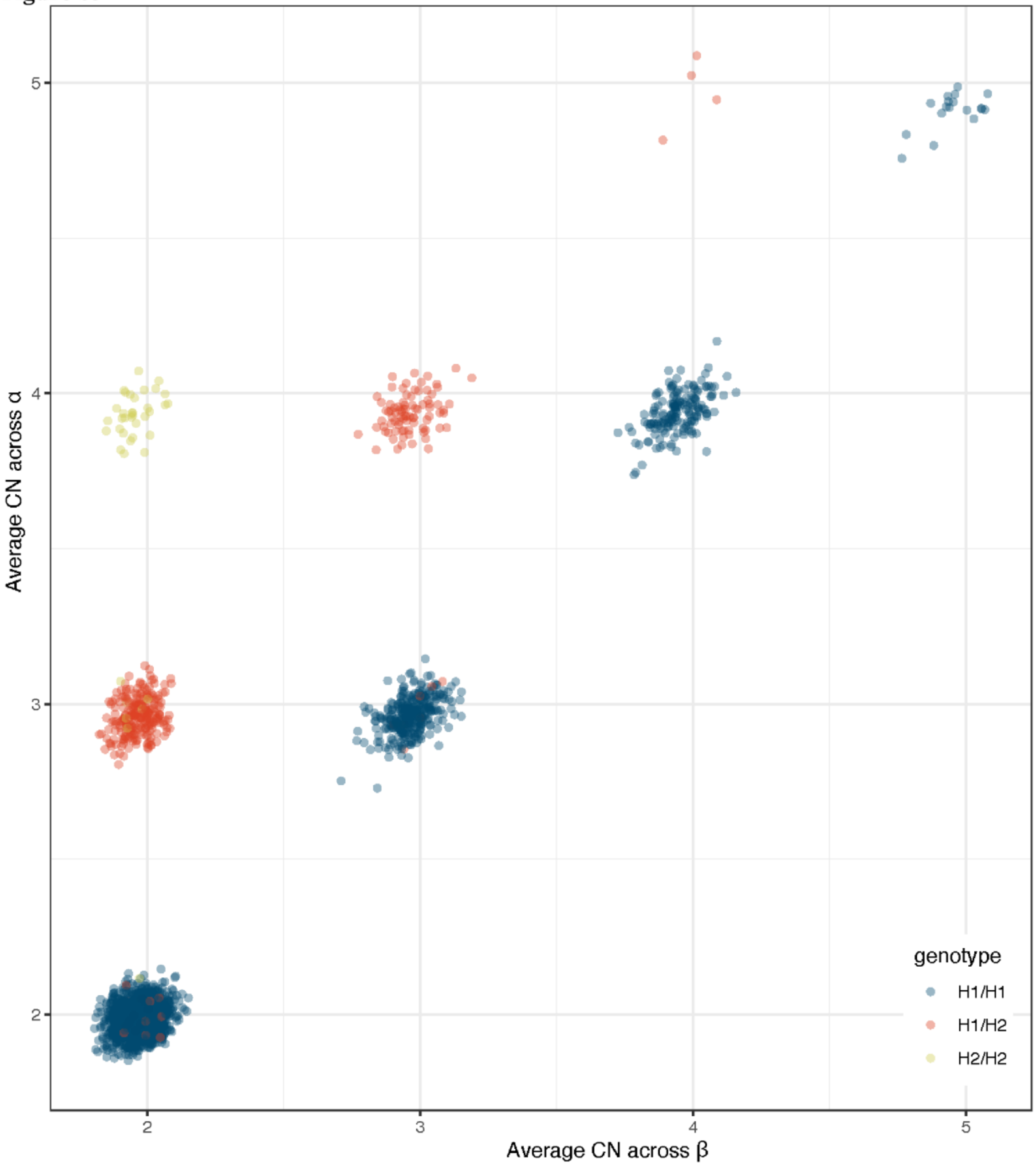

Supplementary Figure 3 – Inversion genotype assignments mapped atop copy numbers in the β and α regions: Each point represents an individual with the x-axis denoting the normalized read depth across β and the y-axis denoting the normalized read depth across α. Points are colored according to inversion genotype, assigned using 1271 tag SNPs. Jitter was applied to both the x and y axes to improve visualization of overlapping points.

Figure S4

	H1.β1	H1.β2	H1.β3	H1.β4	H2.α1	H2.α2	H2.α3
H1.β1	2,2						
H1.β2	3,3	4,4					
H1.β3	4,4	5,5	6,6				
H1.β4	5,5	6,6	7,7	8,8			
H2.α1	2,2	3,3	4,4	5,5	2,2		
H2.α2	2,3	3,4	4,5	5,6	2,3	2,4	
H2.α3	2,4	3,5	4,6	5,7	2,4	2,5	2,6

Supplementary Figure 4 – Table assigning complex genotype status based on α and β regions: Cartesian coordinates (x,y) represent complex genotype assignments with numbers indicating the average copy number as discrete values. The value before the comma corresponds to region β, and the value after the comma corresponds to region α. Red cells denote ambiguous cases which can only be distinguished based on 1271 tag SNPs that are required to differentiate between inversion status. Blue cells represent the most conservative assignment which was used in ambiguous cases within the direct or inverted haplotypes.

Figure S5

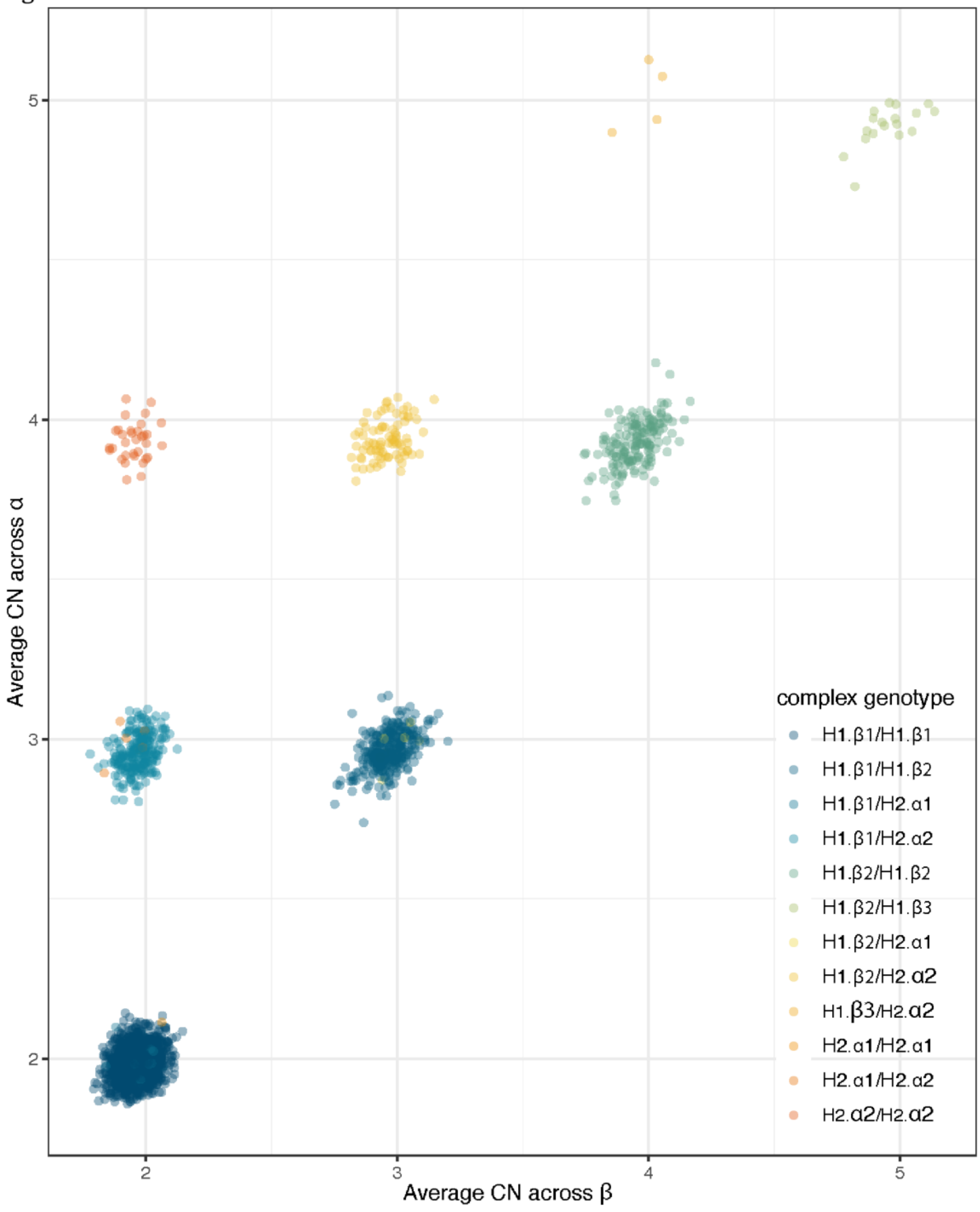

Supplementary Figure 5 – Complex genotype at the *KANSL1* region mapped atop copy numbers in the β and α regions: Each point represents an individual with the x-axis denoting the normalized read depth across β and the y-axis denoting the normalized read depth across α. Points are colored according to complex genotype status, assigned based on the *KANSL1* region using the criteria described in Supplementary Figures 2 and 3. Jitter was applied to both the x and y axes to improve visualization of overlapping points.

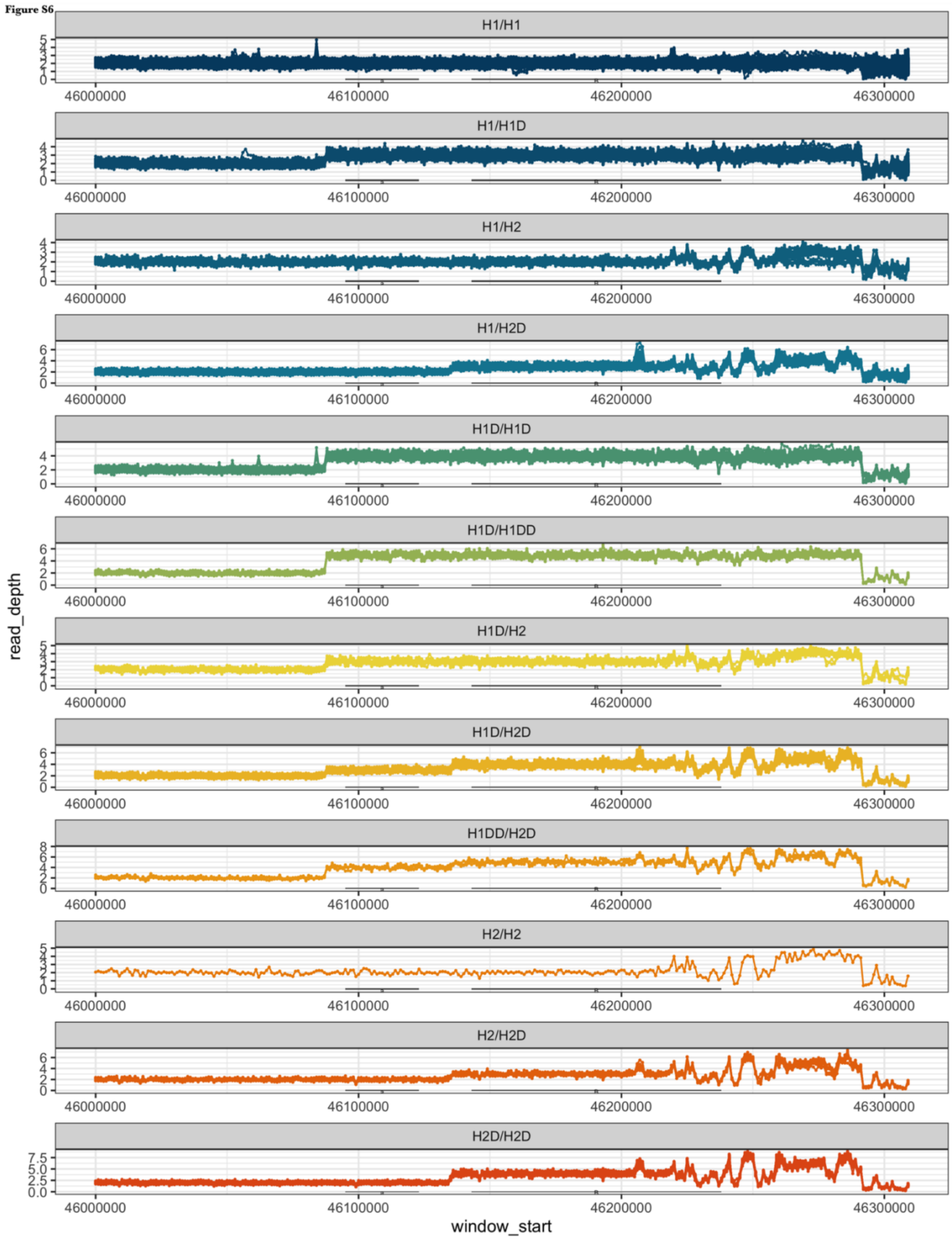

Supplementary Figure 6 – Read depth across the 17q21.31 locus split by CNV assigned by *KANSL1* calls: Each line represents an individual colored and grouped by complex genotype. The x-axis denotes genome position, and the y-axis shows the average normalized

read depth calculated in sliding windows of 1000 bp. Grouping by complex genotype enables visual comparison of CNV patterns across complex genotypes. Horizontal grey lines at the bottom of each plot indicate the positions of the α and β regions, highlighting the differences in read depth between complex genotypes. This visualization complements the discrete copy number genotype assignments shown in Supplementary Figures 3 and 4, illustrating the underlying read depth variation that defines α and β.
