# Supplementary material for: Recurrent structural variation and recent turnover at the 17q21.31 locus in humans and great apes
Source: Nat Commun. 2026 May 19;17:6568. doi: 10.1038/s41467-026-73174-1 (PMC13381761; doi:10.1038/s41467-026-73174-1)
Supplement: Supplementary file 2 — Description of Additional Supplementary Files [file 41467_2026_73174_MOESM2_ESM.pdf]

Description of Additional Supplementary Files

Supplementary Data 1 : Inversion tagging SNPs

Supplementary Data 2 : Frequencies of H1.β1, H1.β2, H1.β3, H2.α1, and H2.α2 in 1000 Genomes, HGDP, SGDP and CAAPA2 samples

Supplementary Data 3 : Frequencies of diploid *NSF* copy numbers in 1000 Genomes, HGDP, SGDP and CAAPA2 samples

Supplementary Data 4 : Recombinant individuals and recombinant breakpoints in 1000 Genomes, HGDP, and SGDP samples

Supplementary Data 5 : Frequencies of H1.β1, H1.β2, H1.β3, H2.α1, and H2.α2 in Ancient European Genomes

Supplementary Data 6 : Long read sequencing alignments for all analyzed sequences in the HGSVC and HPRC datasets, including query and target coordinates, strand orientation, alignment length, annotation notes, and final filtering decisions.

Supplementary Data 7 : Structural haplotype label derived from PGRTK bundle annotations.
