# Supplementary material for: Recurrent structural variation and recent turnover at the 17q21.31 locus in humans and great apes
Source: Nat Commun. 2026 May 19;17:6568. doi: 10.1038/s41467-026-73174-1 (PMC13381761; doi:10.1038/s41467-026-73174-1)
Supplement: Supplementary file 4 — Transparent Peer Review file [file 41467_2026_73174_MOESM4_ESM.pdf]

nature portfolio

Peer Review File

Recurrent structural variation and recent turnover at the 17q21.31 locus in humans and great apes

Corresponding Author: Professor Peter Sudmant

This file contains all reviewer reports in order by version, followed by all author rebuttals in order by version.

Version 0:

Reviewer comments:

Reviewer #1

(Remarks to the Author)
This manuscript describes a detailed genomic analysis of the complex structural variation at 17q21.31 in humans and great apes, and makes inferences on evolution across different timescales. It uses primarily existing data, including pangenome and ancient genomes, together with some new genomes from great apes. It collates this information and reanalyses extensively the data together, building on previous work but providing an important step forward for understanding this locus. I do not need persuading of the importance of this type of work, as complex structural variations a re challenging and detailed understanding of them on a locus-by-locus basis is often needed to understand their evolution and their biological consequences. In particular, the signs of recent possible recent positive selection are very interesting. However, this locus has been repeatedly associated with several complex traits in robustly-powered GWASs. Because of the complex structural variation at this locus, and the high LD resulting from recombination suppression in inversion heterozygotes, fine-mapping putative causative alleles at this locus is challenging, and, to my knowledge, has not really been done with a very high degree of certainty, yet. This uncertainty has limited functional characterisation of these alleles. By providing a detailed picture of the variation at this locus, and a set of well-characterised references on the Pangenome, and other samples, this paper provides an important resource to those who wish to fine map causative alleles and conduct functional assessment. In turn, these functional assessments, as well as informing disease studies, will feed back into ideas on the putative selective force for the KANSL1 duplication.
I have no serious concerns with this manuscript. It is clear, well-written, the methods are well-established and applied appropriately. Interpretation of the data is reasonable and does not go beyond what the data supports. I enjoyed reading this.
A couple of suggestions:
Line 82 – (cite) should be removed
Supplementary tables – it would be very useful to have a reference list of Pangenome haplotypes and ancient genomes with the full SV inferred described in the H-alpha-beta-gamma nomenclature. This would make building on this work much easier.

(Ed Hollox)

(Remarks on code availability)
n/a

Reviewer #2

(Remarks to the Author)
Herein Sridharan et al characterize the 17q21.31 locus through analyses of multiple large datasets, including the human pan genome consortium data and telomere to telomere assemblies of primates. The author detail the structure and evolution of this region by performing state of the art analyses that are clearly described. Overall I enjoyed reading the manuscript, and it represents the latest analysis of 17q21.31, however it shares many similarities with other papers exploring similar complex regions of the genome, such as the 22q11 region (https://pubmed.ncbi.nlm.nih.gov/40631282/). The manuscript is overall well written, but there are multiple typos and other formatting issues.

Major comment
The authors perform a variety of analyses using short-read sequencing data. Particularly to detect inversions tagged by SNPs. Could they have detected the inversion directly using software such as pangenie?

Figure 3C: the text is too small to read. Can the haplotypes be indicated by some colour instead?

Minor comments

Line 82: complexity of this region. We first used PGRTK 23 (cite)
Remove "(cite)" and add the appropriate citation.

Line 99-105:
The text appears greyed.

recently assembled chimpanzee and bonobo genomes (Rocha et al. in prep)
don't mix citation styles.

Line 55: and recurrent structural rearrangement ("toggling")
please describe the concept of toggling in more detail, as its used throughout the manuscript.

Line 243: structural variation can be detected from short read-based SNPsin some cases -> SNPs in

(Remarks on code availability)
The code is available at https://github.com/sudmantlab/17q21.31_SV_and_evolution, the repository is lacking readme files (most of them are empty documents). Some scripts also contain hardcoded paths that needs to be adjusted.
Better documentation would be needed to make this code useful to the readers.

Reviewer #3

(Remarks to the Author)
In the manuscript "Recurrent structural variation and recent turnover at the 17q21.31 locus in humans and great apes", the authors present a comprehensive study on the evolution of an inversion variant in the genomes of humans and their relatives. Overall, the methodology is sound, the results are interesting, and the conclusions are justified. I have only minor comments:

1) I don't think the statement "Gorillas may be at risk for Koolen-de-vries syndrome" (line 139) is meaningful. Either there is evidence that symptoms similar to human patients with this syndrome are common in gorillas, or on the genetic background of gorillas this variant does not have a drastic effect.

2) I don't think any information is given as to the origin of the chimpanzee individuals used in this study. Given an estimated time of 330 kya for the inversion specific to this lineage, it would be interesting to see whether it is found only in certain subspecies (if different subspecies are contained in that unpublished dataset), or not.

3) I'm not convinced by the assumption of positive selection on the H1beta2 haplotype in Europeans. The time transect used in this study overlaps with massive migration events from the Near East and from the Steppe, both of which contributed to genetic diversity in Europe. This is beyond just complex demographic history, since source populations of these migrations could have had a high frequency and contributed substantial amounts of ancestry to subsequent European populations. This might be an easier explanation than selection, unless an explicit model of combined processes is provided.

4) Is there a reason archaic hominins (which are in between great apes and within-human variation on evolutionary timescales) were not considered here? Are the ancient genome pipelines applied here not applicable to Neanderthals and Denisovans? After all, it would be very interesting to see their status at this locus.

5) For the windowed PCA, it would be useful to know if a filter on number of observed SNPs per window was applied, or how many SNPs were observed, on average, since 10kbp blocks are rather short.

(Remarks on code availability)

Version 1:

Reviewer comments:

Reviewer #2

(Remarks to the Author)

I find that the authors have responded to comments and that the manuscript is suitable for publication.

(Remarks on code availability)
Overall the bioinformatic analyses are well explained in the method section. I did not try to install or rerun the code.

Reviewer #3

(Remarks to the Author)
My points have been addressed appropriately, and I have no further concerns.

(Remarks on code availability)

Open Access This Peer Review File is licensed under a Creative Commons Attribution 4.0 International License, which permits use, sharing, adaptation, distribution and reproduction in any medium or format, as long as you give appropriate credit to the original author(s) and the source, provide a link to the Creative Commons license, and indicate if changes were made.
In cases where reviewers are anonymous, credit should be given to 'Anonymous Referee' and the source.
The images or other third party material in this Peer Review File are included in the article's Creative Commons license, unless indicated otherwise in a credit line to the material. If material is not included in the article's Creative Commons license and your intended use is not permitted by statutory regulation or exceeds the permitted use, you will need to obtain permission directly from the copyright holder.
To view a copy of this license, visit https://creativecommons.org/licenses/by/4.0/

We thank the reviewers for their positive feedback and were encouraged by their overall strong interest in the manuscript noting its "comprehensive" nature, "sound methodology," and overall "important step forward for the understanding of this locus." The reviewers highlighted several minor comments which we have addressed in the point-by-point response below.

Reviewer #1 (Remarks to the Author):

This manuscript describes a detailed genomic analysis of the complex structural variation at 17q21.31 in humans and great apes, and makes inferences on evolution across different timescales. It uses primarily existing data, including pangenome and ancient genomes, together with some new genomes from great apes. It collates this information and reanalyses extensively the data together, building on previous work but providing an important step forward for understanding this locus.

I do not need persuading of the importance of this type of work, as complex structural variations a re challenging and detailed understanding of them on a locus-by-locus basis is often needed to understand their evolution and their biological consequences. In particular, the signs of recent possible recent positive selection are very interesting. However, this locus has been repeatedly associated with several complex traits in robustly-powered GWASs. Because of the complex structural variation at this locus, and the high LD resulting from recombination suppression in inversion heterozygotes, fine-mapping putative causative alleles at this locus is challenging, and, to my knowledge, has not really been done with a very high degree of certainty, yet. This uncertainty has limited functional characterisation of these alleles. By providing a detailed picture of the variation at this locus, and a set of well-characterised references on the Pangenome, and other samples, this paper provides an important resource to those who wish to fine map causative alleles and conduct functional assessment. In turn, these functional assessments, as well as informing disease studies, will feed back into ideas on the putative selective force for the KANSL1 duplication.

I have no serious concerns with this manuscript. It is clear, well-written, the methods are well-established and applied appropriately. Interpretation of the data is reasonable and does not go beyond what the data supports. I enjoyed reading this.

> *We thank the reviewer for their positive comments.*

A couple of suggestions:

Line 82 – (cite) should be removed

> *We have made this correction.*

Supplementary tables – it would be very useful to have a reference list of Pangenome haplotypes and ancient genomes with the full SV inferred described in the H-alpha-beta-gamma nomenclature. This would make building on this work much easier.

We have now included these data in supplementary tables 5 and 7.

Reviewer #2 (Remarks to the Author):

Herein Sridharan et al characterize the 17q21.31 locus through analyses of multiple large datasets, including the human pan genome consortium data and telomere to telomere assemblies of primates. The author detail the structure and evolution of this region by performing state of the art analyses that are clearly described. Overall I enjoyed reading the manuscript, and it represents the latest analysis of 17q21.31, however it shares many similarities with other papers exploring similar complex regions of the genome, such as the 22q11 region (https://pubmed.ncbi.nlm.nih.gov/40631282/). The manuscript is overall well written, but there are multiple typos and other formatting issues.

We thank the reviewer for their positive comments. We have now cited the 22q11 manuscript and additionally have proofread the manuscript extensively.

Major comment
The authors perform a variety of analyses using short-read sequencing data. Particularly to detect inversions tagged by SNPs. Could they have detected the inversion directly using software such as pangenie?

While pangenie could indeed be used to detect some of the structural haplotypes we observed, it also has some drawbacks. Specifically we were able to genotype many more structural haplotypes than are present in existing long read assemblies. This is because we employed both tag SNPs (which would likely be captured by pangenie's kmer approach) and read depth over specific loci. Taken together, while pangenie is a very useful tool, because we focussed on deeply assessing a single locus we were able to perform more fine grained genotyping than pangenie would enable "out of the box."

Figure 3C: the text is too small to read. Can the haplotypes be indicated by some colour instead?

We have added a key and removed labels from each of the haplotypes to improve clarity.

Minor comments

Line 82: complexity of this region. We first used PGRTK 23 (cite)
Remove "(cite)" and add the appropriate citation.

We have made this correction

Line 99-105:

The text appears greyed.

We have made this correction

recently assembled chimpanzee and bonobo genomes (Rocha et al. in prep)
don't mix citation styles.

We have made this correction

Line 55: and recurrent structural rearrangement ("toggling")
please describe the concept of toggling in more detail, as its used throughout the manuscript.

We have added a further description of the "toggling" term.

Line 243: structural variation can be detected from short read-based SNPsin some cases -> SNPs in

We have made this correction

Reviewer #2 (Remarks on code availability):

The code is available at https://github.com/sudmantlab/17q21.31_SV_and_evolution, the repository is lacking readme files (most of them are empty documents). Some scripts also contain hardcoded paths that needs to be adjusted.
Better documentation would be needed to make this code useful to the readers.

We have updated the github to provide improved documentation.

Reviewer #3 (Remarks to the Author):

In the manuscript "Recurrent structural variation and recent turnover at the 17q21.31 locus in humans and great apes", the authors present a comprehensive study on the evolution of an inversion variant in the genomes of humans and their relatives. Overall, the methodology is sound, the results are interesting, and the conclusions are justified. I have only minor comments:

We thank the reviewer for their positive comments and feedback.

1) I don't think the statement "Gorillas may be at risk for Koolen-de-vries syndrome" (line 139) is meaningful. Either there is evidence that symptoms similar to human patients with this syndrome are common in gorillas, or on the genetic background of gorillas this variant does not have a drastic effect.

The microdeletion that leads to Koolen-de Vries syndrome in humans occurs very rarely even among individuals that carry the H2 haplotype that is predisposed to this microdeletion. The chance of observing such a deletion in the highly limited set of gorilla genomes, or disease phenotypes in small populations of wild or captive gorillas, is therefore exceedingly low. The fact that Koolen-de Vries-like symptoms haven't been reported in gorillas is thus not evidence for the microdeletion not having an effect or that the haplotypes are not sensitizing. We feel it is appropriate and important to note the presence of such potentially sensitizing haplotypes in gorillas, which may provide clues to veterinarians about the types of symptoms to look for. In fact, early work from Sudmant et al. did identify a chimpanzee individual with a microdeletion that causes Smith Magenis syndrome in humans (Sudmant et al 2013, Genome Research), and only then was this individual properly diagnosed despite showing characteristic symptoms, as Smith Magenis syndrome was not known to occur in chimpanzees before then. The key point we wish to communicate is the presence of such sensitized haplotypes in the gorilla populations, a topic which has garnered little attention outside of humans. Thus, we feel it is appropriate and important to note the presence of such potentially sensitizing haplotypes in gorillas. We have modified the text to reflect this observation in a more nuanced fashion.

2) I don't think any information is given as to the origin of the chimpanzee individuals used in this study. Given an estimated time of 330 kya for the inversion specific to this lineage, it would be interesting to see whether it is found only in certain subspecies (if different subspecies are contained in that unpublished dataset), or not.

We now clarify the subspecies of all chimpanzee genomes and highlight that these haplotypes were observed in two western chimpanzee individuals (*Pan troglodytes verus*).

3) I'm not convinced by the assumption of positive selection on the H1beta2 haplotype in Europeans. The time transect used in this study overlaps with massive migration events from the Near East and from the Steppe, both of which contributed to genetic diversity in Europe. This is beyond just complex demographic history, since source populations of these migrations could have had a high frequency and contributed substantial amounts of ancestry to subsequent European populations. This might be an easier explanation than selection, unless an explicit model of combined processes is provided.

We agree that we do not provide sufficient evidence to conclude that positive selection is the driving force behind increased frequency of H1β2 haplotypes, however, we attempted to be very careful not to make this conclusion, placing all text speculating on selection in the discussion. To clarify this further we now added text explicitly noting that an alternative hypothesis is demography.

4) Is there a reason archaic hominins (which are in between great apes and within-human variation on evolutionary timescales) were not considered here? Are the ancient genome

pipelines applied here not applicable to Neanderthals and Denisovans? After all, it would be very interesting to see their status at this locus.

> We analyzed three high-coverage Neanderthal and a high-coverage Denisovan individual and found they were all H1.β1/H1.β1 individuals and not particularly interesting. We thus decided not to focus on these archaic individuals. We have added this as a note in the methods.

5) For the windowed PCA, it would be useful to know if a filter on number of observed SNPs per window was applied, or how many SNPs were observed, on average, since 10kbp blocks are rather short.

> The mean number of SNPs per window was 284. Our PCA result shows that individuals cleanly cluster into three groups in most windows, suggesting that we have enough resolution to genotype the inversion in 10kbp windows. We now make note of this in the methods.
